# A Parapraxis for Our Times: A Proposal Designed to Address Our Current Failure to Recognize and Specify Clinical Differences and the Threat to the Training and Future of Psychoanalysts it Poses

**DOI:** 10.1177/00030651261418018

**Published:** 2026-02-28

**Authors:** David Tuckett

**Affiliations:** David Tuckett, Robert Schuman Emeritus Professor of Decision-Making, University College London; Visiting Fellow, European University Iity Institute, Florence, Italy; and Distinguished Fellow, British Psychoanalytical Society

**Keywords:** training, pluralism, Freud, criteria

## Abstract

“Pluralism” makes it difficult for psychoanalysts to know how far what they do in their consulting rooms is different or not to what others who call themselves psychoanalysts do. A consequence is that it is now hard for “psychoanalysts in training” to know what they are supposed to do, if they want to practice “psychoanalysis.” What will a psychoanalytic training give you that other therapists don’t have? This paper responds to these questions and the challenge Alan Sugarman has posed by suggesting a solution is available. In the age of pluralism, Institutes can plausibly provide high-quality psychoanalytic training if they clarify transparently their intentions and relate them precisely to Freuds’ defining criteria of procedure, method and theory. Recent research into defining what psychoanalysts do will be presented. If used, they would permit each institute to state publicly which way of doing psychoanalysis they have adopted in which to train their candidates. Informed and disciplined competition between institutes would then become possible alomg with clear criteria for graduation for “analysts in training.” Better and more transparent guidelines for adequate practice could follow (with potential gains for outcome research). Patients would also be enabled to know what to expect.

Is the idea that psychoanalysts disagree on most aspects of psychoanalytic theory and practice, but nonetheless agree on the need for rigorous standards in psychoanalytic training, a paradox? Or, provocatively, might the apparent paradox be better understood as an emerging parapraxis—an enigmatic eruption from our collective unconscious protesting at the so-far hidden incoherence of claiming to train to high standards when we don’t agree (and they often don’t know) what we are training would-be psychoanalysts to do?

Freud’s (1898/1962) idea was that a parapraxis reveals a hidden conflict. If brought to light it can be managed directly, if there is readiness to take on the emotional and intellectual challenges of enquiry. In this paper, therefore, I want to treat Alan Sugarman’s question to his panel of authors as a prescient request to start documenting some important differences in contemporary clinical practice that are currently glossed over and to use that knowledge to specify what Freudian psychoanalytic practice looks like and what “analysts in training” need to learn to do if they want to practice psychoanalysis.

Elsewhere (Tuckett, 2026) I have summarized arguments in the literature and presented research data to attest that today’s psychoanalysis is marked not only by widespread differences in theory and technique but also by widespread lack of realization of the fact that key terms and practices now mean quite different things to one psychoanalyst compared to another. I have also suggested that it may have been appropriate before his death to rely on Freud’s authoritarianism. And I think it not unreasonable that pluralism developed as a necessary corollary to the attempts of several of his successors to build new dynasties based on claims to be heirs to his authority. However, I have also argued that the infinitely tolerant pluralism that has evolved over the past 80 years into “anything goes” is unsuitable and perhaps unethical for an accountable and transparent modern profession.

In sum, for me, behind what I am calling Sugarman’s sensitive parapraxis is a conflictual idea waiting to become crystalized. If in future there is to be a clinical discipline meaningfully and rigorously called “psychoanalysis,” current institutional solutions to defining it locally by democratic voting rather than attempting to agree and publish explicit transparent criteria, need to be confronted.

As already mentioned, Freud saw parapraxes as manifest signs of latent conflict. In the next section I will begin by elaborating on the way Freud defined psychoanalysis using three principles he linked indivisibly—a procedure, a method and a body of theory developed specifically from the findings emerging from the first two (Freud, 1923/1955b, p. 235). Thereafter, I will explore evidence that there is significant but not fully recognized conflict between what Freud meant by “psychoanalysis” and much current psychoanalytic clinical practice.

Using the framework and findings of a recently published study of how ordinary psychoanalysts practice psychoanalysis (Tuckett et al., 2024), I will describe some details as to how the clinical practice of significant numbers of contemporary psychoanalysts differ from each other and from Freud. I will then propose that we might usefully recognize a range of practices consistent with Freud’s proposal and label them as Type Z psychoanalytic practice. Other practices, temporarily defined as “not Z” but better defined in detail by their adherents, will be differentiated as Type X.

Types Z and X are intended as Ideal Types in Max Weber’s sense and constructed using Freud’s (1923/1955b) threefold definition of psychoanalysis spelling out the objectives, principles and ways of implementing them that follow. Freud’s threefold definition brought together procedure, method and theory in a logical and therefore restrictive way. Nonetheless, I want to make clear that Type Z remains quite a broad definition, Potentially, it encompasses much practice in Britain, France, Latin America, and the United States although there are substantial differences. My purpose in specifying Type Z is to challenge all those who wish to train psychoanalysts, to spell out, in relation to Freud’s definitions, exactly what they are trying to provide. I refer here to what an Institute might advocate as intentionally the same or different to Freud’s model as well as to the need to spell out supporting theories and, where it exists, the reasoning behind any shift.

For example, and to anticipate, does an institute train candidates to use Freud’s procedure of free association and evenly hovering attention as a normal setting for everyday psychoanalytic sessions? If not, what variations are advocated and for what reasons? A debate about differences framed in that kind of way could open the way to recognizing differences much more adequately than at present and so to debating the underlying conflicts in a professional and constructive way.

As this contribution will appear in the *Journal of the American Psychoanalytic Association*, I will orient my focus to North America. The issues identified are, however, certainly not restricted to the North American continent.

## What did freud mean by doing Psychoanalysis?

As mentioned briefly above, when Freud invented the term *psychoanalysis* and defined it in his “Two Encyclopedia Articles” (Freud, 1923/1955b),^1^ he proposed it had three integrated components. He wrote,

Psycho-Analysis is the name (1) of a procedure for the investigation of mental processes which are almost inaccessible in any other way, (2) of a method (based upon that investigation) for the treatment of neurotic disorders and (3) of a collection of psychological information obtained along those lines, which is gradually being accumulated into a new scientific discipline. (p. 235)

In the text he is referring (a) to the special investigatory *procedure* he invented for use in regular sessions to discern what is unconscious in another person, which is otherwise almost inaccessible; (b) to his idea that the procedure he invented is at one and the same time also a *method for* the treatment of neurosis, and (c) to the body of *theory* emerging to explain the data obtained from the procedure. So why did he think all three components are needed to define what we are doing is psychoanalysis?

A starting point for understanding Freud’s position is his self-analysis.^2^

Dissatisfied with his abilities and his hypnosis technique for understanding the unconscious templates creating his neurotic patients’ symptoms and (on and off!) worried about imposing his own theories on them, he took the extraordinary step of, so to speak, turning aside from his work with patients to see what he could find out about his own unconscious. To do it he made his whole person the object of enquiry, hypothesizing he could use his own dreams as data to try to discover his unconscious beliefs and impulses.

In *The Interpretation of Dreams*, he sets out how he discovered some details of his own unconscious life by ignoring the direct meaning of the dream stories he could remember and instead forcing himself to associate to their various discrete elements so as to sense the latent issues. The method described was (after first writing down his dreams) to register in writing the thoughts and feelings that *turned up* in the associations while, quite crucially as he explained, adopting an attitude of neither judging nor rejecting any of them. So, he tells us, he had a dream, wrote down his associations, and then later “guessed” (*erraten*) at the latent, otherwise inaccessible and consciously unwanted meanings. The underlying theory was that changes in context, feelings, details, etc., (formally condensation and displacement) constituted the “dreamwork,” which hid the latent content while simultaneously expressing it.

Although he was somewhat discreet in his written reports, there can be no doubt that this “self-analysis” of his dreams made him aware of powerful emotionally charged latent ideas and uncomfortable fantasies (often linked as he came to see it to the development of his ideas about his and other’s sexuality) that had hitherto remained out of his consciousness (i.e., were inaccessible to him). These ideas and impulses were by their nature embarrassing and discomforting. He theorized that the dream was a way to hold on to them and even to enjoy them but not acknowledge it—which is why there is resistance to them being known. Important to note here is that Freud clearly knew “resistance” to free association and what he was to call “ambivalence” to the process of finding out one’s own unconscious, very personally.

As well as writing all this in his *Interpretation of Dreams* (Freud, 1900/1953b, 1900/1953c), he also reported it as it was happening to his mainly passive correspondent, Fliess, as recorded in their correspondence (Masson, 1985), and, crucially, as he did so, he became very excited by it and the new “truths” he thought he was uncovering (see Anzieu, 1986).

As he became convinced about his own mental processes, Freud now transferred the principles of his method of “self-analysis” to his consulting room, abandoning hypnosis and initiating a formal procedure for sessions with his patients. It consisted of a “fundamental rule” of free association (*freier einfall*) for them and another one, evenly hovering attention (*gleichschwebende Aufmerksamkeit*) for himself—and for those to whom he later taught psychoanalysis.

In his new procedure a psychoanalyst, he argues forcibly, should not listen as in an ordinary conversation or medical encounter nor follow in a normal way. The procedure, he wrote,

rejects the use of special expedient (even that of taking notes). . . . It consists simply in not directing one’s notice to anything in particular and in maintaining the same “evenly hovering attention” . . . [in this way] we avoid a danger which is inseparable from the exercise of deliberate attention. For as soon as anyone deliberately concentrates his attention to a certain degree, he begins to select from the material before him; one point will be fixed in his mind with particular clearness and some other will be correspondingly disregarded . . . in making this selection he will be following his expectations or inclinations. This, however, is precisely what must not be done . . . if he follows his expectations he is in danger of never finding anything but what he already knows; and if he follows his inclinations he will certainly falsify what he may perceive. (Freud, 1912/1958, pp. 111–112)

What needs to be stressed here, above all, is that Freud was not naively believing that it was possible for a patient to say everything nor that the analyst’s attention, preconceptions or biases would not become engaged (see also Lear, 2019). Rather, by creating two normative attitudes to be aimed at, he was setting up a procedure to note divergences when signs emerged in himself or the patient—parapraxes, discomforts, taking sides, and so on.

A further point concerns Freud’s deep thinking about what was “data.” Here, what he wrote makes clear that, for him, the meaning that we attach to our experience is shifting all the time according to context. For example, a person we know and remember can inspire hateful memories one minute and loving ones the next. Which is so? Such ideas make memories a complex phenomenon so that further down the page from the passage just quoted about evenly hovering attention, we find Freud stressing the tendency for the meaning of memories to shift through time: “It must not be forgotten that the things one hears are for the most part things whose meaning is *only recognized later on*” (Freud, 1912/1958, p. 112). Here Freud is building on his 1896 letter to Fliess (Freud, 1896/1985)^3^ and anticipating later discoveries about how memory processes in the human brain work and why. Memory is not designed for simple archival purposes (Schacter & Addis, 2007; Schacter, Addis, & Buckner, 2008). Rather it is there both to improve how we feel and think in the present and to allow time travel (Suddendorf & Corballis, 1997, 2007) in the service of facilitating successful future-oriented action. Retroactive changes in meaning as context and emotions shift are not signs of inaccuracies but fundamental capacities, as well understood now in cognitive and affective science. Sensing all this Freud gave the German word *Nachträglichkeit*^4^ a central role in his theory.

In any case, as Freud concluded,

the rule of giving equal notice to everything [*gleichschwebende Aufmerksamkeit*] is the necessary counterpart to the demand made on the patient that he should communicate everything that occurs to him without criticism or selection [*freier einfall*]. . . . If the doctor behaves otherwise, he is throwing away most of the advantage which results from the patient’s obeying the “fundamental rule of psychoanalysis.” (Freud, 1912/1958, p. 112)

I insert the German terms in the text above and elsewhere because English translation and usage, as well perhaps as the contemporary commonality of “therapy sessions” of all sorts, have tended to dull our understanding of just how revolutionary Freud was. In German the terms *freier einfall* and *gleichschwebende Aufmerksamkeit* both have passive constructions—ideas *fall into* doctor and patient’s minds, as Freud’s associations fell onto the page for later reflection in his own “self-analysis.” In short, the thinking behind the procedure Freud has in mind for a psychoanalytic session is one designed to allow images, thoughts and feelings to *fall* into the patient’s and the analyst’s mind to become the core data. That seems less likely if you are trying “to explain things” to each other or are anxious or unwilling to allow silence and thoughtfulness. As he put it, free association happens analogously to the way a passenger in a railway train notices things passing by the window, which is a very different conception than a psychiatric interview or having a conversation with a professional “about things” such as relationships, complaints, happenings so as to make points or express opinions.

Moving on now from the first term in Freud’s definition of psychoanalysis, the *procedure* (defined as *freier einfall* and *gleichschwebende Aufmerksamkeit*), to the second term, the *method*, through which neurotic patients are successfully treated.

What he is stressing in the second point of his definition is that psychoanalysis, as a method of treatment, works through the participation of both parties who together undertake an *investigation*, using the procedure he designed, whose purpose is to reveal a patient’s currently activated unconscious ideas and impulses. The rationale is that in a “psychoanalytic” session, given *freier einfall*, the patient’s hidden beliefs and impulses push forward to be present in the moment (we might say the activated unconscious facts are present in the moment) so that they are potentially “betrayed” (*verraten*) to the analyst, because he is attending in evenly hovering attention (*gleichschwebende Aufmerksamkeit*), noticing parapraxes and signs of discomfort such as anxiety (i.e., resistance noticed potentially in analyst or patient). Session by session the analyst is then enabled to sense and guess (*erraten*) what would otherwise be inaccessible (i.e., held unconscious to preserve current comfort) and to make interpretations (i.e., comments) aimed not at cognitive understanding but at deepening and elaborating the investigation.

His idea was that his procedure converges with his method (of cure) and he referred to this as the *junktim* (Vassalli, 2001). Importantly, but with perhaps uncomfortable implications, Freud makes clear as he elaborates his position that he sees the procedure and so the method as a process involving unconscious to the patient to unconscious to the analyst cognition, or transmission of meaning (see Tuckett et al., 2024, pp. 117–119). An important point here is that Freud is not separating what might be thought of as a procedure of discovery (listening to the patient and inferring unconscious content) from treatment (telling those discoveries to the patient)—something confused by the way he wrote his case histories in which what he selected to convey was designed to convince the reader.

The idea that interpretations are not treatment in the sense of dispensing expert knowledge and wisdom may be confusing insofar as many modern (and especially professionally trained) students of psychoanalysis come to his ideas with already well-formed ideas. My impression is that very often they expect as psychoanalysts to be dispensing interpretations in a talking “treatment” (see Denis, 2008; Vassalli, 2001). Vassalli (2001) makes the point that Freud’s *junktim* of procedure and method, as just set out, as a practice for psychoanalysts belonged to a wider set of medical philosophical conceptions that would have been informing Freud.

In his day, Freud’s idea that his method was also a treatment would have been consistent with Aristotle’s use of the Greek word *techne*—a word with wide ramifications which cannot be translated by the modern word “technical” precisely because that word implies a separation between what is being done and how it is being done. *Techne*, does not make that separation. In short, the meaning of Freud’s *junktim* proposal is that an interpretation is an intervention designed to advance the investigation, rather than one designed to dispense any wisdom gained after listening, which is a very different notion.

Finally, the third element of Freud’s definition of psychoanalysis stresses the idea that a psychoanalytic session is constituted by a general set of theories (a metapsychology) *installed in the psychoanalyst’s mind* and influencing his listening and practice. These theories he saw as those that have emerged from the consensus findings from past sessions *conducted with the procedure and implementing the method* by all psychoanalysts to date.

In other words, for Freud, procedure and treatment form an indivisible tripod with theory. As to what theories he had in mind, he explicitly refers in the following text of his 1923 encyclopedia articles to the theories that first emerged from the findings of his own analysis and then sessions with patients which for him became “Corner-Stones of Psycho-Analytic Theory,” namely,

The assumption that there are unconscious mental processes, the recognition of the theory of resistance and repression, the appreciation of the importance of sexuality and of the Oedipus complex—these constitute the principal subject-matter of psycho-analysis and the foundations of its theory. (Freud, 1923/1955b, p. 247).

“No one who cannot accept them *all* should count himself a psycho-analyst,” he added.

## Contemporary Psychoanalysis Evolving from Freud’s Psychoanalysis

The extent to which ideas as to how to do psychoanalysis have evolved since Freud can be grasped within the literature from proposals such as those questioning free association as a procedure (e.g., Schachter, 2018) or from criticisms questioning

the epistemological difficulties with analytic neutrality and abstinence, the “problem” of the analyst’s subjectivity . . . the differences between a drive conflict model and a relational conflict model, and the differences in our view of intrapsychic life formed from within each perspective. (Davies, 2018, p. 652)

A full review is beyond my scope here—as is a review of how Freud’s procedure outlined above got turned into something so literal as that implied by “analytic neutrality and abstinence” or the provision of “insight” to the patient.

One way to capture at least some elements of how contemporary psychoanalytic practice has evolved since Freud is to use a new clinical theoretical framework for comparing the ways psychoanalysts work that my colleagues and I have described elsewhere (Tuckett, 2025, 2026; Tuckett et al., 2024) and which came out of the Comparative Clinical methods (CCM) project (Tuckett et al., 2008). From the many comparative distinctions that might be made the new framework selects five main issues on which to focus, namely, (a) the procedures analysts use to create what they suppose are psychoanalytic sessions; (b) the data they draw on from those sessions to infer unconscious mental content; (c) how they suppose transference templates reveal themselves in sessions; (d) what they suppose are the principal sources of patients’ difficulties; and (e) how they suppose, as psychoanalysts, what they do makes a difference.

If we use these five variables to look at Freud’s way of doing psychoanalysis, as just discussed, we can note that he supposed that psychoanalytic sessions were created principally by the procedure characterized by *freier einfall* and *gleichschwebende Aufmerksamkeit* through which the analyst could draw on both conscious and less conscious^5^ senses to infer unconscious content from the sessions. He also describes several ways transference becomes revealed in sessions (see below) and how the neurotic patients he saw had difficulties explained by unconscious mental conflicts (hidden by resistance) linked to partial resolutions of infantile sexuality and the Oedipus complex. The psychoanalyst makes a difference, he supposed, as just discussed, by creating an investigation informed by a state of mind that had absorbed the theory and using his procedure.

Trying to make sense of the data obtained from about 300 specially constructed workshops, Tuckett et al. (2024) described a great variety of practice. Those presenting sessions of their work to other psychoanalytic colleagues were experienced psychoanalysts, often training analysts, coming from all parts of the global community. They were not a formal sample so precise quantification would be meaningless. But substantial numbers of these colleagues were practicing in quite different comparable ways, as judged by the five criteria just mentioned. Important and quite easily discernible differences, for instance, could be seen concerning the procedure they set up, the data on which they drew to make inferences, the way they thought transference templates were revealed, the suppositions they made about the troubles their patients were experiencing and how they supposed, as psychoanalysts, they made a difference. Such conclusions rest on asking in the workshops and after what have now been consolidated as Eleven Questions (Tuckett et al., 2024, p. 237). They are each directed to describing practice in the sessions discussed in terms of the five points just mentioned—procedure, data, how transference templates reveal themselves in sessions, the principal sources of patients’ difficulties, and how psychoanalysts make a difference.

### Procedure

Among the sessions we explored in the CCM groups we could distinguish two main approaches to the procedure the analysts set up. One type, we can call formal, the other type, we can call informal or conversational. These are ideal types in Max Weber’s sense, which means that they describe strong tendencies which differentiate the two.

The first type is broadly marked by the features Freud described as his “procedure.” It was typical “classical” technique as described in North America by Loewenstein (1958) or Loewald (1960, 1979). It would include at least some routine use of the “fundamental rule” for patients, the analyst attending in some sort of evenly hovering way and occasional “interpretations.” A neutral stance was described by Anna Freud (1937) as trying to observe and participate in a session from a place equidistant to Id, Ego, and Super Ego.

The second type, at least in part, has evolved from the first, particularly in the last 40 years and, arguably, as a response to the difficulties psychoanalysts using the first type experienced when they attempted to do psychoanalysis with patients from a wider range of social, cultural and pathology backgrounds.

In simple terms, as patients were found to be less simply neurotic and from less highly educated or socially advantaged groups, perhaps presenting with more narcissistic or borderline (etc.) difficulties, the formal method needed an adjunct. The switch can be recognized in the literature (but has probably happened in many places quite naturally) when authors like Greenson (1965) argued that a “therapeutic alliance” had to be established before “psychoanalysis” proper was possible, or when others, like Loewald (1979), argued that although psychoanalysis was “a distinct and unique therapeutic method, in actual practice [a psychoanalyst] makes use, if sparingly, of [other] therapeutic measures.” These he described as “in themselves not analytic, while inspired and guided constantly by the model of the psychoanalytic method” (p. 158). Loewald elaborated to explain that what he had in mind were “so-called educational measures” by which he meant, “at times encouragement and reassurance.” “If used judiciously,” he wrote, such measures “often make possible and enhance the more strictly psychoanalytic interventions, and this not only in the initial phases of an analysis.”

Today, literature, as summarized in the masterly survey by Gabbard and Western (2003), demonstrates how these additional measures have been expanded. For example, many psychoanalysts now emphasize the relational aspect of therapeutic work and use what they see as the easier to establish space opened by an ostensibly ordinary conversation as a vehicle toward gaining connectedness to their patients and insight into and interpreting unconscious themes for discussion.

To establish which type is in use, formal or informal, we can use one of the Eleven Questions Tuckett et al. (2024) developed. It asks, “Have I created a formal or more informal more conversational procedure to give my patients an opportunity to observe and recognize the thoughts and experiences that fall into their minds in their sessions? How and Why?”^6^

In the workshops as many as half those presenting work could be described as using a formal procedure (e.g., Louise and Georgina in Tuckett et al., 2024, pp. 108*ff*) with the other half using the more informal procedure (e.g., Lesley and Lucie [p. 100] and Gilbert and Claudia [p. 102*ff*]). The same question was also applied to clinical reports from Kurt Eissler (1953), Wilfred Bion (1950/2014), Otto Kernberg (1979), Fred Busch (2013), and André Green (2000), all of whom seemed to use a formal procedure, and to reports by Donnel Stern (2019) and Philip Bromberg (2000), two relational analysts, who seemed to use the more informal or conversational approach.

### Data Used for Inference

In presentations to the CCM groups psychoanalysts were using three levels of data to draw inferences about the unconscious issues in sessions and/or in the patient’s mind.

The first level is the patient’s associational content—the things said in this or another session to which the analyst provides an alternate (unconscious) meaning. Freud’s work is full of such transformations as are many psychoanalytic clinical, papers. Classically, the transformation is done by noticing and giving meaning to things like parapraxes, word choice, associations to dreams or noticing repetitive relational patterns or links to history, and so on. Data at this level was widely used in the presentations at our workshops.

The second level of data for drawing inferences that psychoanalysts use is their experience—their subjective responses. We mean by this the thoughts and feelings of which they become aware (in evenly hovering attention) in the sessions and which they sometimes decide are part of their unconscious response to the patient which they can then use as evidence relevant to inferring the patient’s unconscious mental processes present in the session. In the literature Ogden (1997) and Jacobs (1993) are examples. In Tuckett et al. (2024), the cases of Jana and Nana (see especially pp. 48–50, 94–107) or Louise and Georgina (see especially pp. 39–40, 69–70, 71–73) are examples from the workshops. In published work discussed in Tuckett et al. (2024), sessions presented by Bion (pp. 12–15), Kernberg (pp. 9–12), and Green (pp. 15–17) are examples. Bromberg (pp. 18–20) and Stern (pp. 20–22) also use their own thoughts but are less clear as to whether they treat these as deriving from an unconscious response.

Freud does not describe in detail how he worked sufficiently for any certainty but the details he recalls from his patient’s biographies presumably turned up in his mind in sessions. He certainly makes very clear that the analyst’s unconscious is an essential source of data as his associations were in his self-analysis (see Tuckett et al., 2024, pp. 114*ff*) He also explicitly stated, as quoted above, his view that the psychoanalyst who “*behaves otherwise*,” is “*throwing away most of the advantage*” of asking the patient to free associate. It means in essence that Freud wants to emphasize the necessity of the analyst’s deep subjective engagement (and then reflection on it) and that he does not have in mind the kind of objectivity that has sometimes been argued for or against.

The third level of data for drawing inferences is a meta level. Some analysts brought together the patient’s associations and the analyst’s responses to work with, so to speak, a third level which transformed the other two. In doing this they followed the crucial distinction Freud makes when in sessions in which he alternated observing (i.e., evenly hovering attention) and reflection (Tuckett et al., 2024, p. 114).

Louise, just mentioned, is an example of an analyst using formal procedure and all three levels. To summarize, the sessions she had with Georgina (just mentioned) took the form of quite long associations from her patient (sometimes followed by silence) during which Louise became attentive to her own responses, such as feeling swamped by a collapsing sensation, or finding herself remembering things her patient had told her about being placed alone in a clinic with an infectious illness when very young. She then reflected on these responses of her own (Level 2) and the dream and other associations her patient told her in the same session (Level 1) to create in reflection a combined picture (Level 3) in her mind of how Georgina was unconsciously feeling there and then with someone (her analyst) whom she felt was not only collapsing but also sucking all the life out of her.^7^

### Transference Templates

In his paper on transference, Freud (1912/1958) wrote that

It must be understood that each individual, through the combined operation of his innate disposition and the influences brought to bear on him during his early years, has acquired a specific method of his own in his conduct of his erotic life^8^—that is, in the preconditions to falling in love which he lays down, in the instincts he satisfies and the aims he sets himself in the course of it. This produces what might be described as a *stereotype plate* (or several such), which is constantly repeated—constantly reprinted afresh—in the course of the person’s life . . . the peculiarities of the transference to the doctor, thanks to which it exceeds, both in amount and nature, anything that could be justified on sensible or rational grounds, are made intelligible if we bear in mind that this transference has precisely been set up not only by the conscious anticipatory ideas but also by those that have been held back or are unconscious. (pp. 99–100)

The meaning of the term *transference* has been much debated. I have quoted Freud in full to make the point that transference, as he conceived it in the mature phase of his work, refers to his core theory that in everyone, there is an unconscious internal template through which their experience is made and responded to. The template, therefore, unconsciously influences their present experience, which includes their experience with their psychoanalyst. Logically and unavoidably, although finessed by Freud himself, as well as by others who regard countertransference merely as a sign of inadequate personal analysis, each psychoanalyst must also experience the patients in his or her practice through their inner template.

Today, broadly, all psychoanalysts accept this theory of inner templates. It also happens to be a theory consistent with much current thinking in neuroscience, such as in the idea of predictive coding (Friston, 2010).

The difference between psychoanalysts as to how they understand, use and interpret is not mainly in their agreement with Freud’s general idea, but in the suppositions they put into action, explicitly or implicitly in their sessions around two issues: first, how they suppose transference is revealed to them in sessions and, second, when, how and why to interpret it. Here we focus only on the first issue—recognition.

Tuckett et al. (2024) identified four types of supposition evident in the way psychoanalysts suppose they recognize transference. As before, each of the types is conceived as an “ideal type” in Max Weber’s sense—an abstraction used methodologically to differentiate meaningful empirical tendencies not necessarily encountered in pure form. Each type is defined and denoted by a metaphor: Theater, Immersive Theater, Cinema, and Dramatic Monologue.^9^

To determine which type best describes a particular analyst’s work requires asking 2 more of the Eleven Questions:

1. **When was transference revealed?**^10^ When my patient was talking to me, was I supposing she was conveying the emotional situation believed to exist between us (as unconsciously configured in her mind by an internal template so that it was me as a person she had cast as fearful, seductive, cruel, fragile, empty, etc.), or was I rather supposing that she was conveying the situation more generally between herself and others (as unconsciously configured in her mind as seductive, cruel, fragile, empty, etc.)?
2. **In whom was it revealed?** If talking about the situation between us or with others, have I wondered whether in some way what I take to be my patient’s picture (whether of me or others) is being created by me? If so, how?

Question 2 distinguishes analysts who seemed to suppose the unconscious script laid down in the past could become visible by noticing consequences *in themselves*—for example that they were getting indifferent or sleepy or irritated and came to see this as the outcome of a response in them to their patient—from those who do not.

Question 1 differentiates whether transference is supposed to be “visible” at this moment, now, or more generally.

Answers to the two questions suggest four types of supposition about how transference is revealed are possible, when these two underlying variables (timing and participation) are combined. They are Cinema, Theater, Immersive Theater, and Dramatic Monologue.

Using “Cinema,” which was by the most common encountered in our workshops, is a way of working in sessions in which, in effect, analyst and patient are two film critics watching films the patient has brought along which are displayed on a screen in front of them, with the analyst making sense of the unconsciously scripted relations the patients internal template is creating. Both analyst and patient may then comment. The discussion might be more or less heated or consensual.

Using “Theater,” the next most common, is a way of working, in which, in effect, analyst and patient are conceived as in a theater watching a live play put on by the patient in front of them in which all the scenes portray in some way at that moment the roles the analyst as a person (as a consequence of free association) is being unconsciously *cast* into from the patient’s internal template.

“Immersive theater” is a variation of theater in which an analyst supposes not only that he is cast by the patient’s unconscious pictures of him and his intentions but that also, on occasion, that he can cast the patient from his own unconscious template. “Dramatic Monologue,” meanwhile, refers to suppositions that in sessions the patient and the analyst have an unconscious regressive relationship in which the unconscious template driving the session is revealed by the impact of the patient’s choice of words and any hidden effects on the analyst’s unconscious.

For the purpose of this paper, the key distinction is between cinema and theater. In the former the analyst is sensing patterns made by the internal template when listening to the patient’s thoughts and memories brought into sessions from the patient’s life (that the patient may also give hints of repeating in the analysis). In the latter the analysts is focused on how the template is being revealed in the words the patient speaks indicating his or her experience of the analyst *now in the room*.

If we return to the Louise–Georgina case, it will be apparent that Louise “hears” her patient talking to her about a template driven emotional situation between them producing discomfort (resistance) in Georgina provoked by her unconscious experience of Louise in the moment by moment of the session. Louise is being represented as a “black hole” sucking the life out of the patient. So, the associations, dreams, and responses to interpretation in this analysis are all supposed by Louise to give clues as to the underlying unconscious template through which Georgina experiences life and relationships. In fact, Georgina’s principal complaint was depression and for some years the sessions had felt so empty and hopeless that Louise nearly gave up. The material reported above finally gave Louise clues as to how Georgina’s unconscious internal template was repetitively creating for her deadly and empty experiences of people (including her analyst) who sucked the life from her. Under Cinema suppositions Louise might have “guessed” Georgina’s experience outside and tried to talk about it. Under Theater, she could see it happening in front of her be sensitized to how drastically it could be influencing the way she was perceived and so limiting her usefulness to Georgina until it was understood by them both. Georgina, who had earlier wondered “what use you are to me in the analysis” could later say “No one has ever sensed before that I really have a hole deep down inside me . . . a sadness” and “I think that that’s why I feel I am looking after my mother out of duty.” In Theater this is also what she had been doing, coming to an analyst out of duty, for years, and to little effect.

Once the CCM group had devised these different ways of understanding how psychoanalysts suppose transference templates are revealed, we looked not only at published cases by well-known analysts but also at Freud’s two main cases—Dora and Ernst (the so-called rat man), which was quite revealing.

As far as the published cases we analyzed were concerned, our analysis suggested that among them Eissler and Busch were most usefully classified as having “Cinema” suppositions whereas Bion and Kernberg seemed to fit “Theater.” Green, also, essentially had “Theater” suppositions but sitting inside suppositions of “Dramatic Monologue.” Interestingly after struggling to place them, we eventually decided Stern and Bromberg were “Cinema.” This latter result was a surprise, as they are both relational analysts who quite clearly work in an immersive interactional way. It means it is important our reasoning is understood and I recognize that might well merit a lot more discussion.

Essentially, our assessment rests on the fact that although Stern and Blomberg worked in highly interactional relationships with their patients, being responsive to their doubts, asking their opinions, trying to take them into account and noticing their emotional responses, which all might suggest Immersive Theater, neither take up whether they were mixed up with their patients via mutually *unconscious* casting. They do not report any details to suggest, for example, that they thought they were being experienced unconsciously by their patients as failed or depressed or “sucking the life out of them” parents in the sessions, or that they had come to conclude they had somehow been unconsciously acting like them.^11^

Elsewhere (Tuckett, 2025) I have described how the two psychoanalysts that Kohut describes as treating his Mr. Z., although presented by him as having two very different approaches based on the theories with which they tried to understand the material, were actually similar in the way they thought transference was revealed in that they both appear to have supposed “Cinema.” Like Stern and Blomberg or, indeed classical analysts, like Eissler, Kohut makes plain that he derives ideas about the transference templates driving repetitive patterns in patient’s relationships from their stories and memories brought into the room rather than by their casting of their analysts evident in the room (Tuckett, 2026; Tuckett et al., 2024, pp. 214–216).

The new framework has also made Freud’s ideas about transference clear in a hitherto not recognized way. It is has long been argued that the crucial breakthrough in Freud’s understanding of transference as the dominant influence in the analytic situation starts to appear in his retrospective understanding that Dora left him as the outcome of his neither understanding nor interpreting her transference-based experience of him (e.g., Bird, 1972). But what has not been seen clearly before is the shift he made in his practice, when in his analysis of Ernst (the Rat Man) he exchanged his intense efforts to try to convince Ernst they were allies in looking at the repetitive problems Ernst brought into the room (qua Cinema).

The key point is that Freud came to realize that he was not experienced by Dora or Ernst as an objective interpreter of what they told him. He also realized his efforts to reassure Ernst that he was a neutral observer had not worked. For instance, when he said to Ernst, that “I myself was not fond of cruelty like Captain N., and that I had no intention of tormenting him unnecessarily” (Freud, 1909/1955a, p. 169), Ernst still doubted the interpretations.

As described, the case only turned round when Freud fully realized, using the comparative terminology, that he and Ernst were not in the Cinema but in the Theater and that progress was being held up because Freud, as far as Ernst was concerned unconsciously, was neither intent on being helpful nor neutral. Rather he was a person with all the attributes of the cruel captain/father who was feared. He was also (unconsciously) suspected, despite all Ernst’s desperate denials that he thought no such thing, of wanting to take revenge on Ernst in the sessions. Only when this was established could things move.^12^

### Patients’ Difficulties

All the analysts who presented in the CCM workshops described in our research (Tuckett et al., 2024) portrayed patients whose early experience had presented them in one way or another with rather severely unbearable impulses and feelings. Georgina, discussed earlier, was one such an example.

But what they supposed about the relevance of this early experience and how it impacted their patients in the present varied.

The discussion of one group of psychoanalysts’ presentations suggested that these analysts predominantly supposed that their patients’ troubles were the outcome of a deficit in their early experience with caregivers that had somehow continued in unmodified form. A deficit in their capacity to feel or symbolize was preventing successful intimate relationships.

Meanwhile, discussions of a second group of psychoanalysts’ presentations suggested that these analysts predominantly supposed that although their patients had also often suffered deprivation, their troubles were principally the outcomes of failures to resolve infantile conflict, particularly in managing love and hate in the Oedipal situation. Such patients’ problems in the present were conceived as the outcome of the unconscious ways they had evolved to deal with inner conflicts evoked in situations with people they want to love and be loved by, specifically the analyst. In other words, in one way or another, they suffered from unconscious ambivalence.

Interestingly, we found that the difference between the two types of analysts just noted were often correlated in their suppositions about internal transference templates and whether they were revealed in the analytic situation in what I have called Cinema or Theater fashion. Analysts of the first type were usually classified as adopting suppositions to which we applied the cinema metaphor, whereas analysts of the second type tended to adopt suppositions to which we had applied either the theater or immersive theater metaphor.

As far as we could tell the two alternative ways (or perhaps emphases) of thinking psychoanalytically about patients’ troubles linked rather precisely to Anna Freud’s (1976) discussion of the widening scope of patients being taken into psychoanalysis and the subsequent debate about whether standard technique should be modified. Her view, stated firmly, was that psychoanalysis was a treatment designed for those whose “Ego-created” defenses were the cause of their problems as compared to other patients with such real ego deficits that they could not be treated by psychoanalysis.

### Making a Difference

The literature on the therapeutic effects of psychoanalysis is vast and so far beyond the scope of this contribution. However, in our research we noticed that there seem to be three discernibly distinct sets of suppositions among contemporary psychoanalysts focused on (b) creating a process in which more or less explicit interpretation of a patient’s unconscious belief templates is the objective; (b) creating what we might call a discussion around the patient’s thinking difficulties and awareness of hidden feelings; or (c) building a relationship with the analyst designed to improve the capacity for relating more generally.

The main finding, in line with the literature, was that contemporary psychoanalysts adopt a wide plurality of approaches and strategies to make a difference, encompassing many aspects of the analyst’s behavior and often going well beyond “the method” using Freud’s procedure. For example, there seems to have been a quite widespread shift toward either relationship-building strategies or toward what analysts seems to suppose are efforts to help patients to articulate their thinking and beliefs. A sign of the latter would be somewhat ambiguous interpretations of the kind “you are telling me that”—in which when analysts intervened, they suggested a different content for what the patient had just been saying. A sign of the former were efforts to establish relationship of trust and understanding in which the analysts tried to be different to figures their patients had experienced in the past. Such shifts away from Freud’s “investigation” method described above correlate with the argument of Gabbard and Westen (2003) that psychoanalysis has multiple modes of therapeutic action.

## Freudian (Type Z) and not Freudian (Type X) Practice

The descriptions offered so far will have made it apparent that psychoanalysis, as practiced today, embraces a very wide range of approaches and that some of them conflict with each other. For example, the idea of an unconscious transference template “turning” object relationships into repetitive experiences is at odds with the idea that an analyst can deliberately be different to past objects.

In any case in line with my remarks at the beginning of this paper, I now want to propose a possible solution to the conundrum as to how Institutes can clarify what sort of psychoanalysis is offered in their training and so develop rigorous ways both to teach it and evaluate their success.

Table 1 aims to represent at least some of the core issues that appear to separate the practices of contemporary psychoanalysts from each other within the framework of Freud’s (1923/1955b) tripartite definition of psychoanalytic practice discussed above.

**Table 1.** Type Z Psychoanalytic Clinical Practice: An Operationalization of Freud’s Tripartite Definition of Psychoanalysis

Features	Objectives	Principles	Implementation	11Q^a^
1. Procedure	Create a formal “Investigation” of almost inaccessible mental processes with frequent regular sessions.	A. Fundamental Rule for the Patient	Inform the patient of the rule and then enable its use.	Q3
		B. Evenly Suspended Attention with technical neutrality for the Analyst	Adopt a stance as discussed by Freud (1912/1958), Bion (Talamo, 1997), Birksted-Breen (2012) or Donnet (2001) – see also Tuckett et al 2024, Tuckett, 2019.	Q3
2. Method	Sustain the Investigation using the procedure by recognizing and trying to reveal unconscious mental processes present in sessions.	A. Recognize signs of unconscious presence.	Know and use as appropriate three data levels.	Q5, Q6
		B. Recognize signs of transference presence—a stereotype template (*ein Klischee* = transference) comprising unconscious beliefs/phantasies *influencing* emotions and associations.	Know and be able to detect the presence of transference templates using any of the four sets of suppositions for revealing it (Cinema, Theater, Immersive Theater, Dramatic Monologue).	Q1, Q2
		C. Interpret presence of transference and unconscious signs sufficient to sustain process.	Use designation and construction,^b^ as appropriate to sustain the investigation.	Q4, Q7, Q10, Q11
3. Theory—Metapsychology	Conduct the procedure after understanding and internalizing the ability to recognize core aspects of theory.	A. Recognize Unconscious Infantile sexual phantasies and conflicts operational in sessions	E.g., Signs of omnipotence, repetitive excitement, domineering, fragility, etc.	Q8, Q9
		B. Recognize Resistance and repression operational in sessions	E.g. Signs of anxiety, discomfort, parapraxis, etc.	Q10

a. See Tuckett et al. (2024, p. 237).

b. For the distinction between designation and construction, see Tuckett et al. (2024, p. 190*ff*) and Tuckett (2019).

In the first column to the left, therefore, are Freud’s three defining features of psychoanalysis: procedure, method and theories (which should suffuse the practicing analyst’s mind as s/he followed the procedure using the method).

In the next column, each of these defining features is elaborated in short form. So, psychoanalysts are conceived to create a procedure that they deem suitable for capturing unconscious mental processes in the patient. They must also have a way to sustain an investigation using that procedure in which signs of unconscious mental processes become revealed in sessions. And they must also conduct their investigation using the procedure in a mental state in which core aspects of psychoanalytic theory (metapsychology) are instantiated in their minds.

Then in the third column (principles) more precise operationalizations as to how each of these objectives are met are set out—the presence or absence of those operationalizations then allows any given example of practice, for instance a session presented, to be considered psychoanalysis of Type Z, or not (in which case, Type X).

The procedure in use is recognized by assessing if the fundamental rule and evenly hovering attention (or any functional equivalent) are used, or not.

The method in use is recognized by assessing how the investigation is being used in sessions to detect (a) the presence of unconscious processes revealed in it (or not), (b) the presence of transference templates comprising unconscious beliefs influencing emotions and associations, and (c) by the presence of interpretations aimed at sustaining the process of bringing unconscious mentalization to the front.

The recognizable theories observed to sustain the investigation using the procedure might be defined as those an analyst is using to recognize transference and resistance and, for instance, the impact of infantile sexual phantasies and conflicts and resistances toward knowing them in sessions. Recall, that as Freud put it, the assumption of unconscious mental processes, recognition of the theory of resistance and repression, and the appreciation of the importance of sexuality and of the Oedipus complex are the principal subject matter of psychoanalysis and the foundations of its theory. So, such theories “should” be instantiated in the analyst.

The fourth column indicates some further detail—for example, the task of recognizing unconscious process probably requires knowledge of (and the ability to use) some or all of the three levels of data to use to draw unconscious inferences that are available in a session. Or, perhaps, the ability to detect transference templates revealed in sessions requires knowledge of the different ways this is done in that institute. Or, perhaps, the ability to make “psychoanalytic” interventions requires knowledge of how to give implicit or explicit designating or constructing interventions. The details, here are not exhaustive. Finally, when to comes to what theory (metapsychology) should be internalized and so in use, the most obvious signs a psychoanalyst will recognize are those of omnipotent mental functioning, repetitive excitement and signs of anxiety, discomfort, parapraxis, and so forth, as well as signs of unconscious participation through enactment by the psychoanalyst.

The final column links the implementation of the principles to the Eleven Questions developed in our research. They have been designed to be highly practical questions to be asked of the last session any would-be psychoanalyst conducted.

In sum, the overall idea of Table 1 is that practices aiming to fall within the scope of the items identified under objectives, principles, and implementation, demonstrate either that a form of Type Z psychoanalytic practice, consistent with Freud’s definition, is present, or not. Practices that are not consistent might be labeled as Type X. And note that as mentioned, there are undoubtedly different ways of doing either Type Z or X which could be further defined.

Type Z practices, although widespread, are certainly *not* the only type of practice claimed to be psychoanalysis that we found being practiced by reputable psychoanalysts in the workshops.

Such alternative forms of practice, Type X, appear to fall into two groups.

First, there are what we might call practices that are *deliberately divergent* from Type Z because they are believed in various ways better—for example those derived from Kohut’s self-psychology innovations (see Tuckett, 2025) or from the interpersonal socially aware approaches of White, Sullivan, or Horney who had specific disagreements with Freud many years ago. In these cases, Type X practices are supported by theory backed therapeutic approaches that arise in one way or another from modifying Freud’s tripartite definition in an intentional way. They are neither inherently superior nor inferior to Type Z practices but *different*.^13^ They require different principles to be understood and operationalized and so different emphases in training and its evaluation. Institutes need to decide, therefore, if they are training to do Type X or Type Z forms of psychoanalysis and advertise and explain their choice.

Second, there are practices which differ from some significant elements of Type Z procedure, method or theory so that they need to be classified as Type X, not due to deliberate choice and an underlying rationale as to why they suppose practice should not follow Freud’s three principles, but rather because practice has evolved in response to exigencies of one kind or another—perhaps principally due to the challenges presented by the widening scope of patients that psychoanalysts have been trying to treat but also due to complex personal reactions such as those Kohut has made evident and which I have labeled an “inadvertent betrayal” (Tuckett, 2025).

A principal purpose of this contribution and Table 1 (which should be considered a preliminary work in progress) is to open the way toward clarifying divergence more rigorously so as to provoke reflection and clarity. If each institute decided to audit in detail where its training practices and objectives stand in relation to Type Z practice, specifying where, if anywhere, it parts company, it would be a huge step forward toward a disciplined and ethical field and it would greatly clarify for candidates what they are trying to learn.

## Discussion

Above, I briefly described how in the North American mainstream the shift from Freud’s tripartite definition of psychoanalytic practice was clearly recognizable, *de facto*, at the time of Loewald’s (1979) paper, although at that time he was at pains to make his suggestions for widening technique *only as an adjunct* to what he called the “distinct and unique method.” Like Kohut (1979), Loewald’s aim was to propose methods for those patients who could apparently not take advantage of the standard one—in other words for patients who were part of the “widening scope.”^14^

I have argued (Tuckett, 2025) that Kohut, who stated he was not out to upend Freud, may have arrived at his proposals to change technique and theory in what is a relational direction and downplaying the importance of Oedipal conflict inadvertently, as a result of two mistaken but hidden assumptions. One was that transference templates are *only* revealed via a “cinema” approach and the other that *only* Data Level 1 (as opposed levels 2 and 3) is available for inferring unconscious content. Freud could make no impression on Dora or Ernst with those assumptions and nor could Louise (above) on Georgina. Dora, Ernst, and Louise unconsciously “cast” their psychoanalysts as (in various ways) opposed to their wellbeing. In the cinema, as Freud tried, the only way to manage such patients is to attempt to be “different.”

A hypothesis to consider, therefore, is that the shift toward relational techniques in the United States, which diverge from Type Z in several ways, is a modern reaction to the unrecognized consequence of Freud’s psychoanalysis as it was imported into North America. The tsk was undertaken at a time in the early 20th century when “science-based” medical professions were in an embryonic stage of development and keen to be differentiated from folk practitioners and quackery of all sorts.^15^ The medical practitioners who discovered Freud were people eager to help their patients and attracted to Freud’s ideas to do so. But, at one and the same time, they were intent on trying to ensure their specialty remained within medicine. They wanted to steer a wide birth from accusations of quackery, at the time of the Flexner report^16^ and afterward. Was this one factor causing the leading theoreticians of the day like Hartmann, Kris, and Loewenstein to formulate Freud’s thinking in a locally familiar and acceptable way? In doing so we can, perhaps, now see that they subtly shifted or left out elements that have been a central focus elsewhere but might have been more vulnerable to criticism in North America—particularly the extent to which Freud’s clinical psychoanalysis relied on subjective factors and unconscious-to-unconscious communication. It took clinical psychoanalysis in a different direction. In particular, it seems to have forced widespread reliance on expecting transference to be revealed “in the cinema,” as I have described it, and using Level 1 data (rather than subjective Level 2) as admissible. A related very literal version of neutrality also took hold with countertransference, for a long time, considered mostly as a sign of insufficient personal analysis.

It appears to me that all forms of psychoanalysis have vulnerabilities (Tuckett, 2026; Tuckett et al., 2024). But psychoanalysis practiced under Cinema and Data Level 1 suppositions is vulnerable in those specific instances in which patients create major subjective responses in their analysts, or where the psychoanalyst (by means of their privileged cultural and socioeconomic location) is distant from the patient’s experience in the outside world (as with intersectional divides (Crenshaw, 1989^17^) or where the patient has internal transference templates, like Ernst and Dora, which give them an *unconscious* belief that their analyst is not trustworthy, kind, or reliably understanding, despite the therapeutic contract and the analyst’s conscious wish to help. One might add that, as in Kohut’s case, these problems are particularly likely to be found in the analysis of would-be psychoanalysts as well as other patients who in many other ways are functioning well.

The hypothesis then is that the shift from the “distinct and unique method,” or what Kohut calls the technique of the “classical analyst,” to self-psychology and relational analysis may have been motivated by a deep belief on the part of analysts, as in Kohut’s case, that the method they had been trained in was “not-working.” But was this belief based on a restricted or even false assumption—namely the assumption that the Hartmann, Kris, and Loewenstein ideas (and their later followers) about transference templates and unconscious inference they had been trained to use, were the only ways to implement Freud (See Tuckett 2026)?

As work by psychoanalysts like Fred Busch (2013) (and see Tuckett et al., 2024, pp. 7–9, 210–211), demonstrate, there is no doubt that Cinema and Level 1 suppositions can work very well. But when the patient has internal reasons for fearing or hating (or for that matter loving and wishing to seduce) their analyst, my clinical and supervising experience suggests to me that the psychoanalyst will inevitably be profoundly emotionally challenged in sessions. At that point, if you have not been taught to value and reflect on your emotional and other responses, or worse still to think of them as your pathology, including feeling imprisoned in anxiety yourself when your patient is profoundly anxious, and are trying to infer transference templates using Cinema suppositions, then the situation can become very painful. It seems possible, as with Kohut, that the resulting desperation could create the impression that Freud’s method (as you understand it) has to be revised, leading to more relational ways of practice.

## Conclusion

The argument I have set out, using the research framework and findings emerging from the CCM workshop research, has aimed at highlighting the possibility that an outcome of pluralism is that many contemporary psychoanalysts have come to practice psychoanalysis in ways which appear to be in marked variance from Freud’s definition of what psychoanalysis should be. Dr. Sugarman’s parapraxis—if I can call it that—is perhaps motivated by a difficult awareness that we cannot go on like this.

I have summarized above a Type Z way of defining psychoanalytic practice that is within Freud’s tripartite definition and have also framed it with sufficient operational content, supported by the Eleven Questions that can be asked of the last session any psychoanalysis has conducted, to be useful. No doubt Type Z could be differentiated into Types Z^1^, Z^2^, and so on, and no doubt different versions of Type X are also possible. No doubt the two types of practice can be developed further but in my view they are already sufficient to provide any individual or group with a framework to discuss and describe the practice they wish to train their candidates to enact, if they can overcome what is likely to be the deep emotional challenge of doing so. It is this emotional challenge, of course, that is suppressed by pluralism (Author, in press-b). And that emotional challenge may be too considerable.

It is perhaps of some interest that currently the Web site of the American Psychoanalytic Association (https://apsa.org/about-psychoanalysis/) is very forthcoming in providing extensive information about various definitions of psychoanalysis and psychoanalytic concepts^18^ and of the training programs offered within its participating training institutes. The latter is not summarized under any shared core principles but was assembled by candidates who have provided an extraordinary amount of detail about the varied programs, requirements and procedures of each Institute, sometimes including curriculum and reading lists. The material runs to many hundreds of pages (American Psychoanalytic Association, 2024) but would not easily allow a candidate to decide whether he or she would get a Type Z or Type X training.

In North America the American Board of Psychoanalysis (ABPsa), has conducted an ambitious and valuable empirical enquiry using focus groups composed of those providing training to would-be psychoanalysts, to establish what were considered core competencies. The results surprised the investigators. They found that although, formally, their respondents came from a very wide range of psychoanalytic traditions with conflicting approaches and diverse goals, the recordings and transcripts…revealed that although different language may have been used, participants in all the groups were really saying the same thing.” In some ways this is a reassuring start but given the variability in practice observed and discussed in the pages above the agreement is also disconcerting. It suggests that when those conducting training try to specify what they are helping “candidates” to do, they could not get beyond very general capacities. The ABPsa is continuing it work and may get more specific.

Meanwhile, If Freud’s psychoanalysis is to stay alive, it seems to me psychoanalysts and their institutes should be deeply be concerned about conceding to “bar-lowering” generality, even if pursuing differences and disagreements to arrive at whether an institute is training Type Z or X psychoanalysts (and exactly how) is challenging, both emotionally and cognitively. There are many forces at work that resist recognizing differences among psychoanalysts—accepting difference after all creates anxiety and opens up rivalry and is one of the deep challenges required to work through the Oedipus complex—but if psychoanalysis as a distinct practice is to survive, the challenge cannot be escaped by more “anything goes” pluralism in our governance. This may be the reason for what I have called the enigmatic eruption, the hidden protest, from our collective unconscious, that I have attributed to Alan Sugarman, for which we should salute him.

## Footnotes

1 A definition, note, written *after* the publication of his major theoretical works (the *Three Essays* and the *Interpretation of Dreams*), his main cases, his technical papers and in the same year as his “The Ego and the Id.”

2 The remarks here rely on my joint research with my fellow authors and particularly that of Michael Diercks (see, Diercks, 2018 Tuckett et al., 2024, pp. 113–119).

3 “The material present in the form of memory traces being subjected from time to time to a rearrangement in accordance with fresh circumstances—to a retranscription. Thus what is essentially new about my theory is the thesis that memory is present not once but several times over, that it is laid down in various kinds of indications” (p. 207).

4 *Nachträglichkeit* has been translated into English as *deferred action or afterwardness*, and in French as *après-coup*, literally after-blow.

5 I use the term *less conscious* because it makes no sense to claim you can know your unconscious directly. What you may be able to do is to guess it from signs of it that you are prompted by.

6 This is a modified (for clarity) version of the question which began as “Have I created a formal procedure to give my patients an opportunity to observe and recognize the thoughts and experiences that fall into their minds in their sessions or am I setting up something more like an ordinary conversation?” (see Tuckett et al., 2024, p. 237).

7 Two more of the Eleven Questions are used here. Question 5 (*attitude to own thoughts and feelings as data*) asks, “Do my guesses about the meaning of my patient’s associations rely to a significant degree, as far as I am aware, on my using my feelings, responses, and thoughts? How?” Question 6 (*reflective curiosity*) asks, “Have I created a reflective setup in my mind to observe and question how I am arriving at my guesses about my patient’s associations and how I am using my own thoughts and feelings? How?”

8 Note, that for Freud, the idea of transference is directly connected to sexual drives and the infantile basis for “love.”

9 Please see Tuckett et al. (2024, pp. 51*ff*) for a full description. To be useful it is important the metaphors are used as defined rather than as terms into which any convenient meaning can be pushed!

10 Again, these questions are slightly modified from those published due to the experience using them.

11 Because the essence of much relational psychoanalysis and its innovative intellectual property, so to speak, is its focus on the relationship between the patient and analyst so that it is, so to speak, highly interpersonal, many readers might suppose that relational analysts would think of themselves (and should be classified as) supposing transference as revealed in an “Immersive Theater.” My view, focusing on clinical accounts like those of Stern and Blomberg (above), is that in fact they work with transference as revealed via “Cinema.” I base this conclusion on finding no evidence in their clinical accounts of attention either to their patient’s or their own possible *unconsciously formed* internal templates, as opposed to their conscious ideas about each other. They certainly do attend to and try to bring out the patient’s conscious and inhibited thoughts or doubts or criticisms. But in doing so patient and analyst appear to be film critics—discussing films of outside relationships or films of sessions. (Some other “here and now” techniques have this feature.)

12 Tuckett et al. (2024) discuss why this shift has not been widely recognized. They argue Freud did not fully internalize or fully recognize his technical shift—really a revolution. Their data also suggests that it is also likely that a majority of today’s psychoanalysts retain the Cinema without realizing approach the Cinema, let alone Immersive Theater possibility.

13 Such judgment would require properly conducted studies of differential outcomes.

14 These issues are enlightened by the debate I was privileged to attend as a young candidate between Anna Freud and Andre Green; see Freud (1976) and Green (1975).

15 Note, administratively, until the Division 39 lawsuit of the mid-1980s opened registration in the International Psychoanalytical Association as a psychoanalyst in North America to psychologists, institutional psychoanalysis was under the control of medically qualified professionals.

16 A report published in 1910 which argued there was a total absence of scientific standards for medical education, clinical practice, and therapeutic devices in the United States. Along with the federal Pure Food and Drugs Act of 1906 the Flexner report changed the course of medicine, and the effects, both positive and negative, are still felt today. The report standardized medical education, supposedly based on scientific principles and demonstrable facts. Practices were declared scientifically unsupportable (although no actual research was done to back up many statements) and legally excluded from clinical practice. Doctors also had certain equipment removed from their offices and taken to “museums of quackery.”

17 Schafer’s (1979) paper on the analytic attitude is of interest here. He noted that the fact that “there is a mutual dependence of the analytic attitude and the psychoanalytically constructed life history should be obvious, neutrality being essential to effective exploration and accurate understanding and vice versa. But beyond recognizing this interdependence, analysts face the urgent task of identifying those additional constituents of the analytic attitude that shape just those life-historical data that are to be understood and organized into a history. Among these constituents are visions of reality . . . and the interpenetrating or correlative investigations of the analysand's past and present actions.”

18 For example, Confrontation, Countertransference, Defense Mechanisms, Denial, Dream, Ego, Ego Ideal, Elektra Complex, Fantasy, Fixation, Id, Libido, Oedipus complex, Parapraxis, Repression, Resistance, and Transference.
